# ESGs and Customer Choice: Some Empirical Evidence

**DOI:** 10.1007/s43615-023-00251-8

**Published:** 2023-01-17

**Authors:** Paraskevi Boufounou, Ιlias Moustairas, Kanellos Toudas, Chrisovalantis Malesios

**Affiliations:** 1 Department of Economics, National & Kapodistrian University of Athens, 1, Sophocleous Str, 105 59, Athens, Greece; 2 Department of Agribusiness and Supply Chain Management, Agricultural Univ. of Athens, 75, Iera Odos Av, 118 55 Athens, Greece; 3 Department of Agricultural Economics and Rural Development, Agricultural University of Athens, 75, Iera Odos Av, 118 55 Athens, Greece

**Keywords:** Sustainable development, ESG indicators, Customer choice, Q01, D12

## Abstract

The goal of this paper is to determine whether a company’s performance on environmental, social, and governance (ESG) indicators influences customer choice, and if so, which ones are the most important, as well as whether the COVID-19 pandemic had an effect on changing this hierarchy. Additionally, it intends to investigate the influence of regional and demographic factors on its formation. To achieve this goal, primary data were gathered in Greece via a questionnaire survey. According to the findings, a company’s performance on ESGs influences consumer choice, with an emphasis on environmental and social indicators. It was also demonstrated that a company’s social indicator performance is relevant to both urban and suburban customers. Customers in urban areas place a higher value on a company’s performance in governance indicators than those in suburban areas, who place a higher value on a company’s performance in environmental indicators. Finally, no significant COVID-19 effect was evidenced on the findings, although the emphasis on “social indicators” was further reinforced, probably due to the increase in social awareness of citizens during the pandemic.

## Introduction

The efforts of modern businesses to maintain momentum in a volatile operating environment are particularly difficult. This is based on the needs of all stakeholders, including employees, local communities, customers, and non-governmental organizations (NGOs). The preceding requirements allude to the need for a responsible and transparent business operation, while also acknowledging the need for the firm to grow and be profitable. Businesses are increasingly attempting to adapt to the application of societal, employee, customer, and environmental protection practices. It is critical for company executives to understand how corporate social responsibility (CSR) actions can be incorporated into their development strategy, risk management, and corporate culture, particularly in the context of sustainable development, which is the new challenge for modern businesses. The UN’s Sustainable Development Goals (SDGs) are a global call to action and initiatives for businesses through their ESG activities. Effective adoption of ESG indicators in conjunction with the implementation of the SDGs is expected to result in benefits for both business and society.

The purpose of this study is to look into the impact of company performance on ESG indicators on customer satisfaction and the formation of consumer preferences in Greece, as well as the impact of geographical factors (allowing for urban and suburban area effects). It also looks into the role of demographic factors in the development of these preferences, as well as whether the COVID-19 pandemic had an impact on them. To accomplish this, this study is structured as follows: first, a theoretical foundation is presented for the concepts of sustainable development, SDGs, and ESGs. Following that is a review of the literature. The research approach is then described, and the key findings are presented. Finally, the main conclusions are summarized and suggestions for practical application are put forward accompanied by recommendations for further research.

### Sustainable Development, SDGs, and ESGs

According to Boufounou and Argyrou [1], the first conceptual definition of sustainable development was presented in 1987 at the United Nations General Assembly, by the Norwegian Minister of Environment Gro Harlem Brundtland, in her report entitled “Brundtland Report,” according to which “Sustainable development is defined as development that meets the needs of the present generation without compromising the ability of future generations to meet their own needs”.

**Fig. 1:**
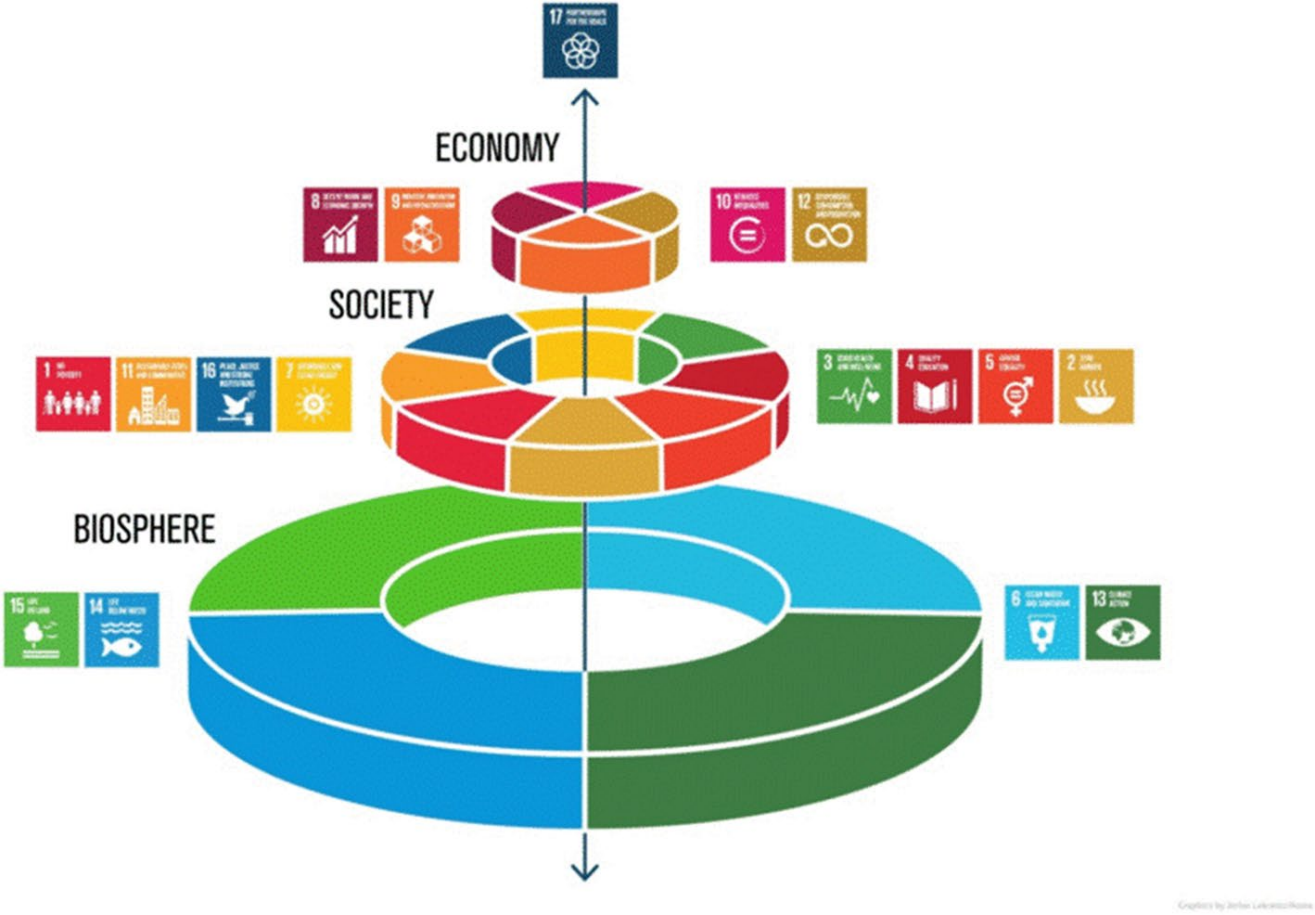
Integration of 17 SDGs across the 3 dimensions of sustainable development. *Source:* Crossman et al. [2], reprinted from Stockholm Resilience Center

The United Nations Member States, acknowledging the crucial role of sustainability, have adopted in 2015 the 2030 Agenda for Sustainable Development. This agenda revolves around 17 Sustainable Development Goals (SDGs), which represents a global and pressing call for action. Figure 1 below provides more information on each SDG.

Moreover, the UN developed the concept of ESG (environmental, social, governance) while working with the financial sector. The main argument was that ESG could shield organizations from financial risks resulting from ESG-related issues as employment disputes, human rights issues, low governance quality, and climate change. More specifically, ESG is comprised of the following 3 factors:

- *Environmental.* These criteria refer to whether the business activity includes actions to protect and manage the natural environment, such as pollutant and greenhouse gas emissions, energy consumption, use of renewable energy sources, climate change mitigation, resource management, and waste management.
- *Social.* They investigate societal, human rights, and labor relations issues in the communities where a company’s operations are located, such as gender equality, equal employment opportunities, pay, employee education and training, community benefits, supplier evaluation, health and safety issues, and data security and privacy issues.
- *Governance*. They examine factors and issues related to management practices, decision-making processes, business ethics, and corporate governance structure, such as executive compensation, corruption, bribery, accountability, and ethics.

The UN started the initiative to incorporate ESG factors into capital markets in 2004. In 2005, the UN released the Compact study, which fundamentally altered the way that people make financial and investment decisions. The Compact study found that incorporating environmental, social, and corporate governance factors into capital markets not only makes good business sense but also ensures sustainable development and yields more favorable outcomes for society as a whole [3]. Recent technological, environmental, and social changes have intensified the demands on the financial industry and capital markets for the incorporation of ESG elements into business operations as well as for the transparency and disclosure of information on these variables [4]. As an example, in 2019, the amount of ESG investment assets under management amounted to $30 trillion [4]. In the context of requirements to integrate ESG factors into business activities and in the transparency-characterized disclosure of their non-financial information, stock exchanges play a crucial role. Stock exchanges can promote the integration of sustainable development in financial activities and encourage transparency in information disclosure by directing capital flows towards sustainable investments and providing incentives for companies with high ESG performance [5, 6].

According to Boufounou and Argyrou [1], the implementation and disclosure of ESGs in Greece is encouraged and initiatives have been taken:

- The Greek Sustainability Code is a practical tool for businesses and organizations that want to promote sustainable development to increase transparency and self-commitment. The Greek Sustainability Code, which is directly linked to the European Sustainability Code, is a structured reference framework that consists of four pillars (strategy, management process, environment, and society) and a total of 20 criteria and presents the level of integration of the principles of Sustainable Development & Corporate Responsibility of organizations at the national level. Based on international reporting standards (Global Reporting Initiative, United Nations Global Compact, the OECD Guidelines for Multinational Enterprises, EFFAS, EMAS), and which meets both the requirements of the European Directive on the disclosure of non-financial information and the needs of measuring the economic, environmental, and social performance of organizations, which aim at upgrading both their image and reputation, continuous improvement, enhancing their competitiveness, positive evaluation by investors and therefore raising capital, joining international “Responsible Supplier Networks,” and strengthening their export activity. To date, 45 Greek companies (the majority of them large) have published reports based on the guide, with the first of them dating back to 2017.
- Participating in the United Nations’ Sustainable Stock Exchanges (SSE) initiative in 2019, ATHEX published the relevant ESG Reporting Guide, the implementation of which is not mandatory. The guide proposes specific key performance indicators (KPIs) for each ESG pillar. The KPIs are divided for each pillar into general, advanced and sectoral [7].
- Furthermore, the new “ATHEX ESG” index began trading on ATHEX in August 2021, and it monitors the stock market performance of ATHEX-listed companies that adopt and publish their performance based on ESG criteria. The current composition of the “ATHEX ESG” index is based on the performance of 35 publicly traded companies, and an annual review is planned.
- Finally, in order to align with Agenda 2030, Greek banks are now incorporating ESG criteria into the process of assessing the creditworthiness/lending capacity of companies, but also, in the near future, of private individuals in terms of mortgage loans based on the energy efficiency of the property. However, there is still insufficient published data in Greece to study these findings. They also create Sustainable Development Management Units at the highest administrative level to measure, capture, and coordinate the impact of their operations on ESGs.

Extant literature has documented the positive impact of ESG practices on firm-level operating and financial performance [8] and on society as a whole. Moreover, research from various markets has shown that CSR activities facilitate customer retention and repurchase intentions (Yu and Tang [9], Tong et al. [10], Jose et al. [11], Pourezzat et al. [12], Saleh, Ebeid & Abdelhameed [13]).

However, in this study, we focus on the Greek market and assess whether a company’s performance on environmental, social, and governance (ESG) indicators influences customer choice. Moreover, we explore which are the most important and whether the COVID-19 pandemic had an effect on changing this hierarchy, thus extending and complementing the extant literature.

## Literature Review

### The Concept of Corporate Social Responsibility

Corporate social responsibility (CSR) has been a top priority for businesses around the world for the last 15 years, as it is regarded as an essential component of the new business model. CSR is defined in the context of evaluating a company’s performance as the company’s acceptance of a commitment to achieve a balance between profits, social welfare, and customer satisfaction [14].

According to Green and Peloza [15], a common definition of CSR cannot be formulated because it is a concept that evolves over time, and this is because consumers perceive CSR business activities as separate initiatives that may enhance their purchase intention from a specific firm, rather than initiatives that shape the overall image of the firm. Rexhepi et al. [16] state that corporate activities have an impact on both the external and internal image of the company, with effects that can be presented in three areas: society, environment, and economy. According to Wolzak et al. [17], CSR aims to achieve profit, taking into account the impact of corporate activities on the environment, society, and internal (human resources) and external (consumers) customers. From the above, it is clear that a high business economic performance, directly dependent on consumer behavior, is the intersection of those corporate activities that aim to protect the environment, reduce social inequalities, and have effective and fair internal governance of the firm [18].

Positive feedback and information (word of mouth) about a firm’s products or services circulated to consumers’ personal communication networks, as well as consumers’ repurchase intention (equivalent to keeping a firm’s customers), are manifestations of their satisfaction [19]. One of the key behavioral outcomes of CSR activities, according to Yu and Tang [9], is consumers’ willingness to speak positively about the companies involved in these social, environmental, and economic activities. Furthermore, Tong et al. [10] and Jose et al. [11] concluded that CSR activities have a positive effect on consumers’ repurchase intentions. Meanwhile, Pourezzat et al. [12] concluded that in the airline services sector, word of mouth dissemination of positive consumer information had a significant positive effect on the repurchase intention of these services. Building on the above framework, the work of Saleh, Ebeid, and Abdelhameed [13] aimed to investigate the impact of CSR activities implemented by specific mobile operators in Egypt on both the dissemination of positive feedback and information from their customers and the retention of these customers. The data collected from 342 consumers in the mobile phone service sector led to the conclusion that CSR activities—and more specifically social and economic activities—have a positive impact on customer retention, with the spread of positive word-of-mouth feedback reinforcing this positive effect.

### ESG Indicators and Their Association to CSR and Customer Satisfaction

A key feature of consumers in the last decade is their growing concern about social, environmental, and ethical issues. The aforementioned growing concern of consumers is considered the main reason for the development and placing on the market of products and services that are characterized as green, ethical, or socially responsible [20]. From the aforementioned, the interrelationship between consumers, CSR, and ESG indicators becomes clear. Moreover, as already mentioned in a previous section, performance on ESG indicators is a criterion for investors. Research in recent years indicates the direct impact of CSR and performance on ESG indicators, on customer satisfaction and therefore on the creation of long-term value and financial performance of each company, criteria that also influence the investors’ decisions [21]. Hornuf et al. [22] pointed out that companies focusing their performance mainly towards social indices in order to achieve increased consumer satisfaction end up attracting more investors. Similarly, the research by Mehta et al. [23] identified a positive relationship between firms’ environmental indicators and investors’ willingness to invest in them in mutual funds.

The aforementioned studies lay down a theoretical framework that highlights how investors’ decision-making is influenced to switch to firms with high performance on ESG indicators and with CSR profiles, aiming for a high financial return directly dependent on customer satisfaction. However, although the majority of research is focused on the relation between ESG indicators and economic performance of companies, there is a lack of research on the topic of investigation of the effects of ESGs on customer satisfaction.

Table 1 below summarizes the main research studies that investigate the link between companies’ performance on ESG indicators and the creation of long-term value for them.

**Table 1:** Literature overview

Authors	Year	Country	No of firms	Goal	Methods	Conclusions
Al‐Najjar and Anfimiadou	2012	Great Britain	350	The impact of companies’ environmental performance and policies on their economic performance and long-term value generation over the 1999–2008 period	Quantitative/Questionnaire	The performance of the companies under review on environmental indicators has a positive impact on both their financial performance and their long-term value creation
Cek and Eyupoglu	2020	USA	500	The impact of ESG indicators on the financial performance of companies	Quantitative/Questionnaire	The performance of the companies under review on ESG indicators has a significant impact on their financial performance, with social and corporate governance indicators being the most critical, compared to environmental indicators
Constantinescu et al	2020	International	70	The impact of ESG factors on the long-term value creation of energy sector companies in 2017	Data compiled by analysts at Thomson Reuters	A positive correlation was found between the performance of the companies under review on ESG indicators and long-term value generation
Dalal & Thaker	2019	India	65	The impact of ESG factors on the profitability of Indian public limited companies calta the period from 2015 to 2017	Quantitative/Questionnaire	High corporate performance on ESG indicators enhances financial performance assessed through accounting metrics as well as market-based metrics
Ionescu et al	2019	International	73	The impact of ESG indicators on the long-term value creation potential of companies operating in the tourism industry over the period 2010–2015	Quantitative/Questionnaire	Corporate governance indicators had the most significant influence on the long-term value of the companies under review
Li et al	2018	Great Britain	350	To investigate whether the disclosure of companies’ performance listed on the London Stock Exchange over the period 2004–2013 affects their long-term value	Data collection and analysis	Public disclosure of ESG performance was positively correlated to long-term value creation
Mishra & Suar	2010	India	150	The impact of CSR activities (in relation to six stakeholder groups—employees, customers, investors, community, environment and suppliers) of companies on their financial and non-financial performance	Quantitative/Questionnaire	Listed companies demonstrate responsible business practices and better financial performance than unlisted companies. Responsible business practices towards key stakeholders can be profitable and beneficial for Indian companies
Mitra & Anas	2021	Indonesia	45	The impact of ESG indicators on customer satisfaction during the Covid-19 pandemic	Quantitative/Questionnaire	Social and corporate governance indicators had a significant impact on customer satisfaction during the Covid-19 pandemic
Monda & Giorgino	2013	France Italy Great Britain Japan USA	100 (listed, highest capitalization)	The impact of corporate governance indicators on long-term value generation over the 2009–2011 period	Quantitative/Questionnaire	The performance of the companies under review on the corporate governance indicators has a positive impact on their long-term value generation
Oprean-Stan et al	2020	Europe	50 (listed, highest capitalization)	Exploring the relationship between corporate performance on ESG indicators and sustainable development in the 2013–2020 period	Collection and analysis of data from companies’ annual reports	Improvements in ESG indicators enhance the sustainable development of companies
Saleh, Ebeid, & Abdelhameed	2015	Egypt	3	The impact of CSR activities implemented by mobile operators in Egypt on both the circulation of positive feedback and information from their customers and the retention of these customers	Quantitative/Questionnaire	CSR activities—and more specifically social and financial activities—have a positive impact on customer retention, with the spread of positive word of mouth reinforcing this positive effect
Yadav, Han, & Rho	2016	USA	394	The impact of environmental performance on long-term business value generation, according to Newsweek’s 2012 “Green Rankings” of major US companies	Quantitative/Questionnaire	The results of companies’ environmental performance, published on the relevant websites (or periodically), influence investors’ decisions. Improving the environmental performance of companies enhances their long-term value
Yoon et al	2018	South Korea	705	The impact of CSR activities on the ability to generate long-term value over the 2010–2015 period	Data collection and analysis	CSR practices have a positive and significant impact on the company’s long-term value generation. However, its effect on share prices may vary depending on the characteristics of the company. For companies operating in environmentally sensitive sectors, the impact of CSR on long-term value generation is lower compared to companies not belonging to these sectors

In particular, Cek and Eyupoglu [24] studied the impact of environmental, social, and corporate governance (ESG) performance on the financial performance of 500 US companies for the years from 2010 to 2015. The authors grounded their research model in the following research hypotheses:

- Companies’ environmental performance has a positive impact on their economic performance. This research hypothesis was based on research papers that found that companies that engaged in environmentally responsible business practices improved their financial performance demonstrated that disclosing greenhouse gas emissions from corporate activities resulted in poor financial performance for these companies [25–28]. The argument linking environmental performance to financial performance is based on the fact that environmental regulations-compliant business operations reduce operating costs, minimize noncompliance fines, and improve the firm’s image in the minds of consumers [25–28].
- Firms’ social performance has a positive effect on their financial performance. The studies on which this research hypothesis is based link social corporate performance to economic performance through issues related to human resource management (social employee management practices are associated with lower turnover, less absenteeism, and increased productivity, all of which improve the firm’s competitive advantage), as well as through activities characterized by social responsibility (human rights, supply chain transparency, quality).
- Companies’ performance on corporate governance indicators has a positive impact on their financial performance. The functions and structure of the board of directors, the remuneration policy, the company’s vision and strategy, and the rights granted to shareholders are all part of a company’s corporate governance structure. Furthermore, companies voluntarily display certain corporate governance elements and information in order to increase transparency [29]. The performance of corporate governance is linked to many indicators of financial performance, such as resource utilization, attracting investment capital, and promoting investor confidence. According to Basdekis et al. [30], there is a strong influence of specific corporate and market features on firms’ profitability in Euro area.
- Monda and Giorgino [31] discovered a link between governance performance and financial performance indicators such as market valuation and return on assets for companies in France, Italy, the UK, and the USA. In addition, when companies in the USA demonstrate improved governance performance, the cost of equity falls [32]. Simultaneously, corporate governance performance improves firms’ ability to pay attention to social issues and consumer demands, both of which contribute to their long-term financial performance [33, 34]. Corporate governance practices help a company’s reputation and image. As a result, directors and CEOs are eager to invest in positively perceived governance-related activities in order to increase company likeability and achieve reputation and prestige [35].

The findings of Cek and Eyupoglu [24], based on the aforementioned literature review, showed that performance on ESG indicators significantly affects the financial performance of these companies under review, with social and corporate governance indicators being the most critical, compared to environmental indicators.

In the same vein, Ionescu et al. [36] investigated the impact of environmental, social, and corporate governance (ESG) factors on the long-term value generation potential of 73 global tourism companies from 2010 to 2015. The corporate governance factor appears to have the most significant influence on the long-term value of the companies examined, regardless of their geographical location.

### The Impact of the COVID-19 Pandemic on ESG Criteria

It is clear from the above overview that CSR and ESG indicators have a positive impact on the financial performance of companies. However, the COVID-19 pandemic has forced companies to rethink how CSR programs are able to contribute to corporate performance, both in terms of profit growth and customer retention and satisfaction, while Basdekis et al. [37] pointed out the existence of high volatility due to the highly uncertain period.

Up to now, this topic remains under-researched in the relevant literature, with only exception of the paper of Mitra and Anas [38]. The aim of Mitra and Anas [38] was to investigate the impact of CSR and ESG indicators. They examined forty-five Indonesian companies’ CSR and ESG indicators on customer satisfaction/retention, long-term value generation, and financial performance before and during the COVID-19 pandemic. Their research findings showed the following:

- Environmental indicators had an impact on the long-term value generation and financial performance of companies before the pandemic event, while during it only on financial performance.
- The social indicators had no effect on customer satisfaction, long-term value generation, and financial performance before the pandemic, while during it they had a significant effect on customer satisfaction and long-term value generation.
- Corporate governance indicators had a significant effect only on financial performance before the pandemic, and during the pandemic on customer satisfaction and long-term value generation.

## Methodology

### Research Approach

The review of the literature in the “Literature Review” section reveals a research gap associated with the lack of current research on the topic of ESG indicators and their association to customer preferences, as well as the additional confounding effects of the COVID-19 pandemic on this relation. Moreover, the literature research identified the absence of potential influence of regional and demographic factors on the aforementioned relationship.

Hence, the present study focuses on examining whether ESG information affects consumer choices and more specifically to examine the following research questions:

- RQ1: Which ESG indicators, and to what extent, influence the consumer’s choice to purchase products/services from companies operating in Greece that implement policies regarding these indicators?
- RQ2: To what extent has the COVID-19 pandemic influenced consumers’ perception regarding the impact of ESG indicators on their choice to purchase products/services from companies operating in Greece that implement policies regarding these indicators?
- RQ3: Are there any differences between the place of residence and other demographic characteristics of the survey participants regarding their choice to purchase products/services from companies operating in Greece that implement policies regarding ESG indicators?
- RQ4: Which ESG indicators are related to and how can they predict customer satisfaction, regarding companies operating in Greece that implement policies regarding ESG indicators?

**Fig. 2:**
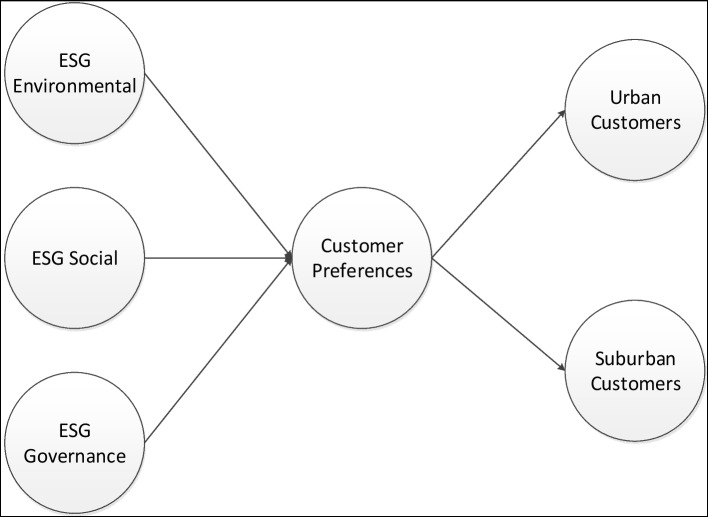
Theoretical model and research framework

The above research questions are the corresponding frameworks utilized for their investigation which are visualized in the following diagram (Fig. 2).

### Data Collection and Construction of the Questionnaire

To accomplish this goal, primary data were collected through random sampling from a Greek population sample of 282 consumers in order to investigate their views and attitudes toward their consumer behavior in general, as well as their current views on ESGs. This is accomplished by developing a relevant questionnaire consisting of 30 questions as follows:

- 5 closed-ended questions to collect demographic data (gender, age, monthly income, educational level, employment status)
- 5 rating scale questions framing the variable “environmental indicators”
- 9 rating scale questions surrounding the variable “social indicators”
- 4 rating scale questions framing the variable “corporate governance indicators”
- 4 rating scale questions framing the variable “customer satisfaction”
- 3 rating scale questions to explore the impact of the COVID19 pandemic on the responses of the survey participants

Grant Thornton [39] studied extensively the sustainable development reporting in Greece and concluded that, although ESG Reporting will become compulsory for listed in the Athens Stock Exchange companies in 01–01-2023, 42% of listed companies already do publish yearly ESG Reports. Furthermore, as specifically stated by Grant Thornton [39] against what was expected, in the random sample examined, only 46% of the companies that published ESG Reports were listed, while the rest 56% were not (of which 22% were SMEs)” and 95% of them upload the ESG Report in their site, while 39% upload it in the social media. In order to investigate the existence of “regional effect” (given the significant differences in terms of social media penetration, growth rates, etc. among regions, that relate to ESG information diffusion), the sample of this study was split into two categories, namely:

- “urban areas” (composed of the responses of 156 consumers in urban areas within the Attica region, where the highest concentration of businesses in the country and therefore the greatest diffusion of ESGs)
- “suburban areas” (composed of the responses of 126 consumers in suburban areas (outside the Attica region)

In addition, as far the “greenwashing phenomenon” is concerned, Grant Thornton [39] evidenced that 75% of all ESG Reports published in Greece until November 2021 were based on GRI Standards and 34% of them were confirmed by relevant qualified external auditors. Modern literature places special emphasis on “greenwashing.” Yu et al. [40] noted that mostly large companies engage and that firm-level governance factors are more important than country factors in deterring greenwashing. Finally, it should be noted that the point made by De Silva Lokuwaduge and De Silva [41] that the diverse approaches to and objectives of sustainability standards and frameworks pose the threat of increasing greenwashing is met at EU level, as the relevant EFRAG Sustainability Reporting Board is going to launch the European Sustainability Reporting Standards that will be followed by all European forms in 2023 onwards (when ESG Reporting will be compulsory for all listed companies, as mentioned above).

The sub-questions framing the ESG indicators were formulated on the basis of ATHEX (2019) [7]. The sub-questions framing the dependent variable “customer satisfaction” were based on Saleh, Ebeid, and Abdelhameed [13] and finally, the questions on the impact of the COVID-19 pandemic were based on Mitra and Anas [38]. The questionnaire was created through Google Forms and was answered anonymously during the period 11/23/2012–18/01/2022 to meet the validity and reliability criteria according to Maxwell [42] and the questions were answered based on the 5-point Likert scale.

### Statistical Analysis

Suitable statistical analysis techniques were utilized for analyzing the obtained data and responding to the research questions of the paper. Specifically, descriptive statistics is deployed in order to summarize indicators and variables collected through questionnaire. In addition, statistical inference techniques such as the Mann–Whitney *U* and Kruskal–Wallis *X*^2^ non-parametric tests were utilized for examining differences in Indicators based on various demographic characteristics. Correlations in the sample were tested using the Spearman’s rho coefficient due to the type of the collected data. Finally, reliability of the sample is tested through the use of the Cronbach’s alpha and inferential analysis for examining the impact of indicators on customer satisfaction was performed through regression modeling.

## Data Analysis Results

The basic characteristics of the sample are described in Table 2 below.

**Table 2:** Descriptive sample data

Demographic characteristics		Urban areas (*Ν* = 156)		Suburban areas (*Ν* = 126)	
		Frequency	Relative frequency	Frequency	Relative frequency
Gender	Male	82	52.56%	72	46.15%
	Female	74	47.44%	54	34.62%
Age	18–25	30	19.23%	26	16.67%
	26–45	62	39.74%	50	32.05%
	46–66	45	28.85%	40	25.64%
	67 and over	19	12.18%	10	6.41%
Income	up to 1000 €	91	58.33%	72	46.15%
	1000–1800 €	46	29.49%	42	26.92%
	1800 € and over	19	12.18%	12	7.69%
Education	Secondary education	44	28.21%	38	24.36%
	Tertiary education	73	46.79%	54	34.62%
	Postgraduate/doctoral degree	39	25.00%	34	21.79%
Occupation	Entrepreneur/freelancer	12	7.69%	20	12.82%
	Private servant	59	37.82%	28	17.95%
	Civil servant	16	10.26%	30	19.23%
	Student	18	11.54%	14	8.97%
	Unemployed	25	16.03%	14	8.97%
	Homemaker	8	5.13%	8	5.13%
	Pensioner	18	11.54%	12	7.69%

With regard to the first research question, the main results of the survey in terms of descriptive statistics are presented below (mean, standard deviation, and graphically), broken down into urban and suburban areas.

**Fig. 3:**
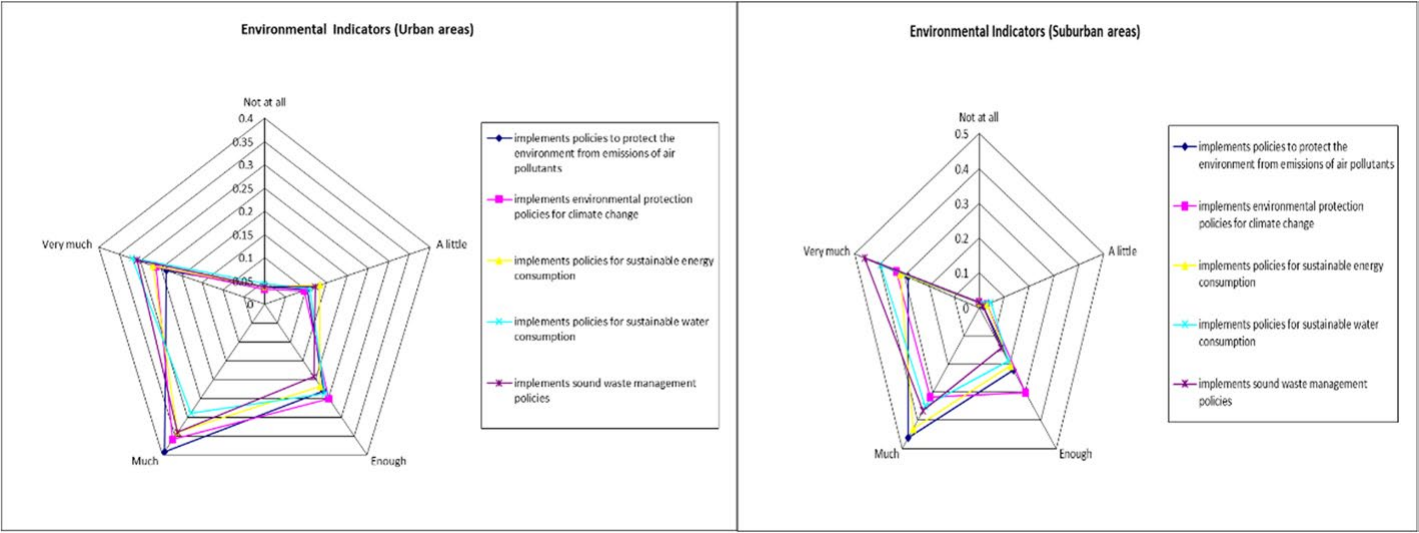
Spider plot of environmental indicators’ results

A summary of the environmental indicators is presented in Table 3 and Fig. 3 below.

**Table 3:** Summary of environmental indicators

	Urban area		Suburban area	
*How important is it for you (from 1* = *not at all to 5* = *very important), when choosing to buy products/services from a company operating in Greece if it:*	Average	Std. Dev	Average	Std. Dev
Implements policies to protect the environment from emissions of air pollutants	3.69	1.06	3.98	0.85
Implements environmental protection policies for climate change	3.72	1.06	3.92	0.95
Implements policies for sustainable energy consumption	3.67	1.13	4.00	0.89
Implements policies for sustainable water consumption	3.73	1.15	4.06	0.96
Implements sound waste management policies	3.76	1.13	4.24	0.87
“Environmental indicators” variable	3.71	1.05	4.04	0.81

**Fig. 4:**
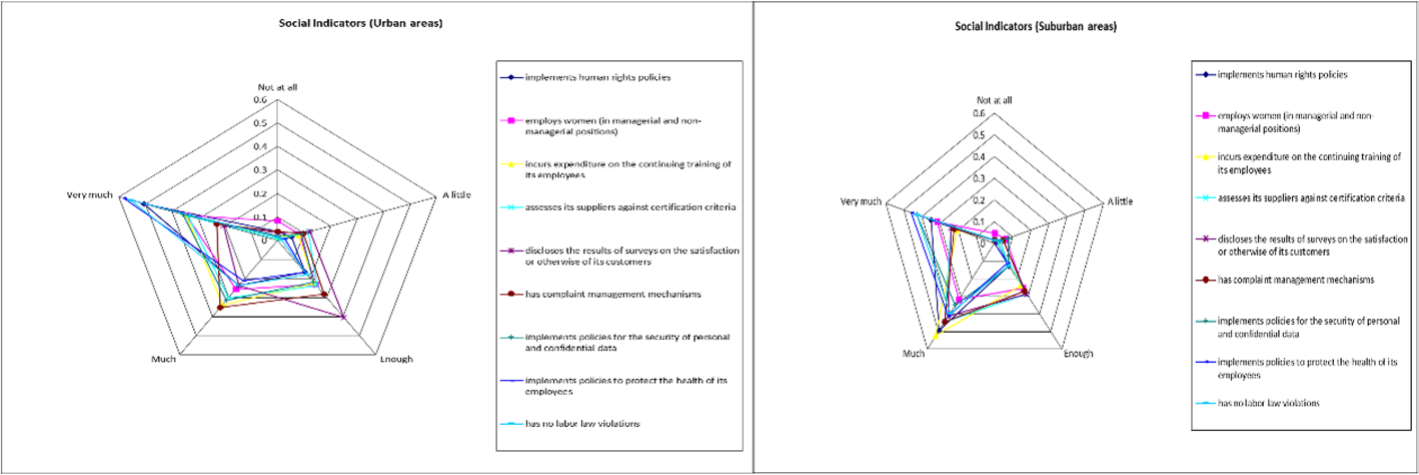
Spider plot of social indicators’ results

Respectively, a summary of social indicators is presented in Table 4 and Fig. 4 below.

**Table 4:** Summary of social indicators

	Urban area		Suburban area	
*How important is it for you (from 1* = *not at all to 5* = *very important), when choosing to buy products/services from a company operating in Greece if it:*	Average	Std. Dev	Average	Std. Dev
implements human rights policies	4.12	1.10	4.14	0.82
employs women (in managerial and non-managerial positions)	3.70	1.26	3.79	1.11
incurs expenditure on the continuing training of its employees	3.93	1.00	3.89	0.80
assesses its suppliers against certification criteria	3.85	1.04	3.87	0.85
discloses the results of surveys on the satisfaction or otherwise of its customers	3.46	1.05	3.81	0.91
has complaint management mechanisms	3.64	1.06	3.81	0.89
implements policies for the security of personal and confidential data	3.90	1.05	4.10	1.01
implement policies to protect the health of its employees	4.33	0.93	4.30	0.79
has no labor law violations	4.31	0.90	4.19	0.89
“Social indicators” variable	3.92	0.87	3.99	0.73

**Fig. 5:**
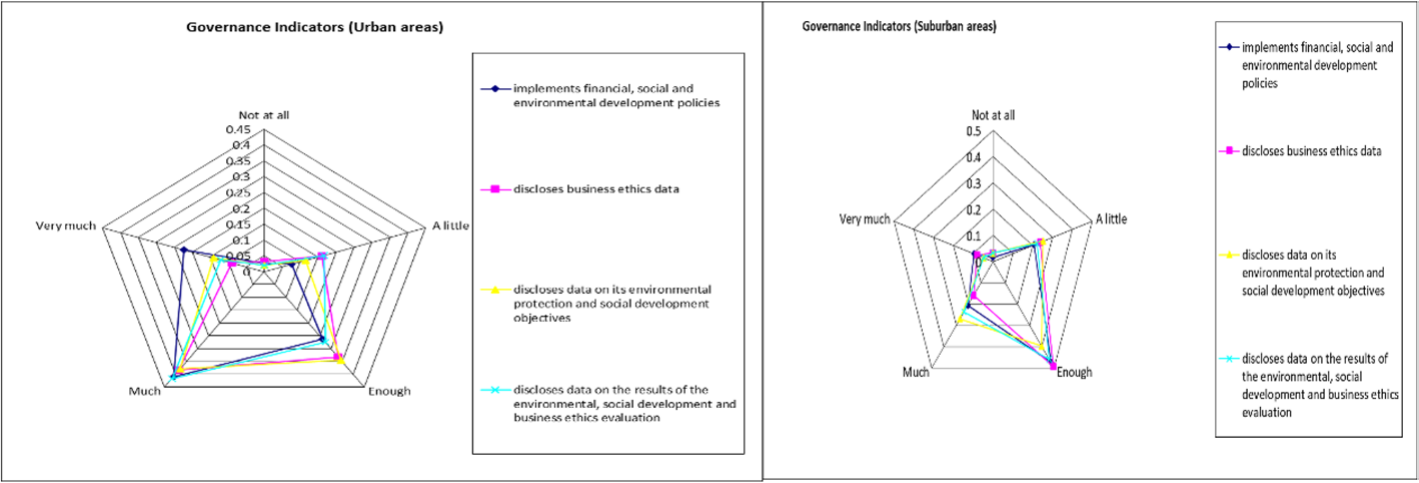
Spider plot of governance indicators’ results

Governance indicators are presented in Table 5 and Fig. 5 below.

**Table 5:** Summary of governance indicators’ results

	Urban areas		Suburban areas	
*How important is it for you (from 1* = *not at all to 5* = *very important), when choosing to buy products/services from a company operating in Greece if it:*	Average	Std. Dev	Average	Std. Dev
implements economic, social and environmental development policies	3.73	0.98	3.16	0.92
discloses business ethics data	3.34	0.96	3.02	0.92
discloses data on its environmental protection and social development objectives	3.51	0.94	3.05	0.92
discloses data on the results of the environmental, social development and business ethics evaluation	3.46	0.97	3.05	0.88
“Governance indicators” variable	3.51	0.89	3.07	0.80

**Fig. 6:**
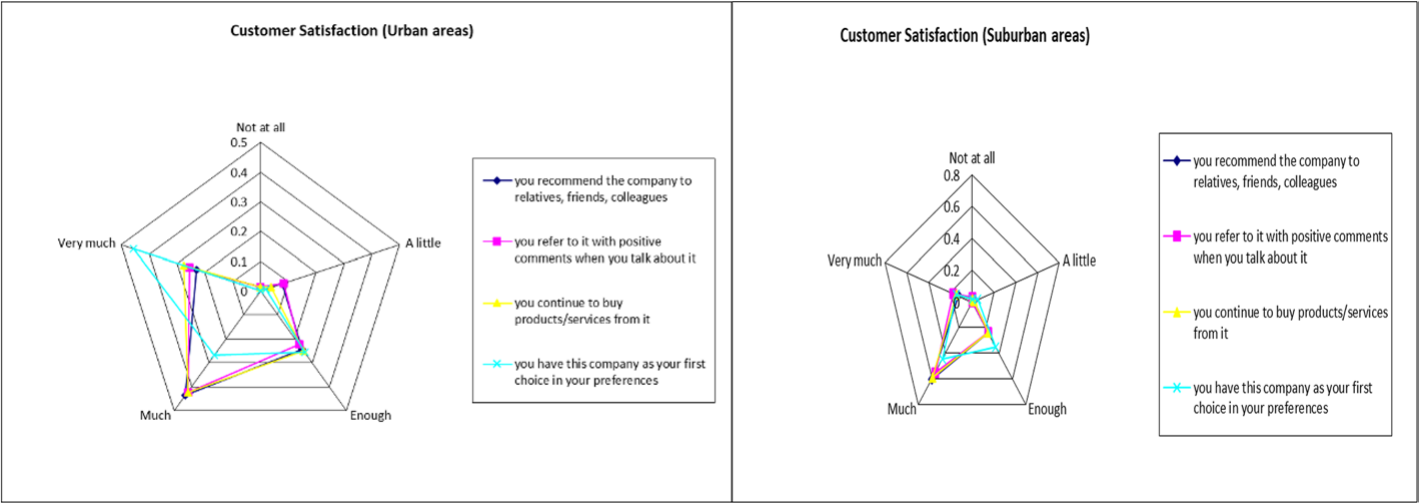
Spider plot of customer satisfaction indicators’ results

A summary of “customer satisfaction” variables is presented in Table 6 and Fig. 6 below.

**Table 6:** Summary of customer satisfaction

	Urban area		Suburban area	
*If a company follows some or all of the above criteria, to what extent (1* = *not at all, to 5* = *very much) are you prepared to:*	Average	Std. Dev	Average	Std. Dev
recommend it to relatives, friends, colleagues	3.79	0.93	3.81	0.73
refer to it with positive comments when you talk about it	3.83	0.95	3.84	0.82
continue to buy products/services from it	3.91	0.89	3.83	0.75
have this company as your first choice in your preferences	3.74	0.88	3.65	0.84
“Customer satisfaction” variable	3.82	0.82	3.78	0.69

It is clear that ESG indicators have a moderate to high influence on consumers’ decisions to purchase products/services from a company operating in Greece that implements ESG policies. Surprisingly, the average values for environmental and social indicators are the highest in both samples of the current survey.

**Fig. 7:**
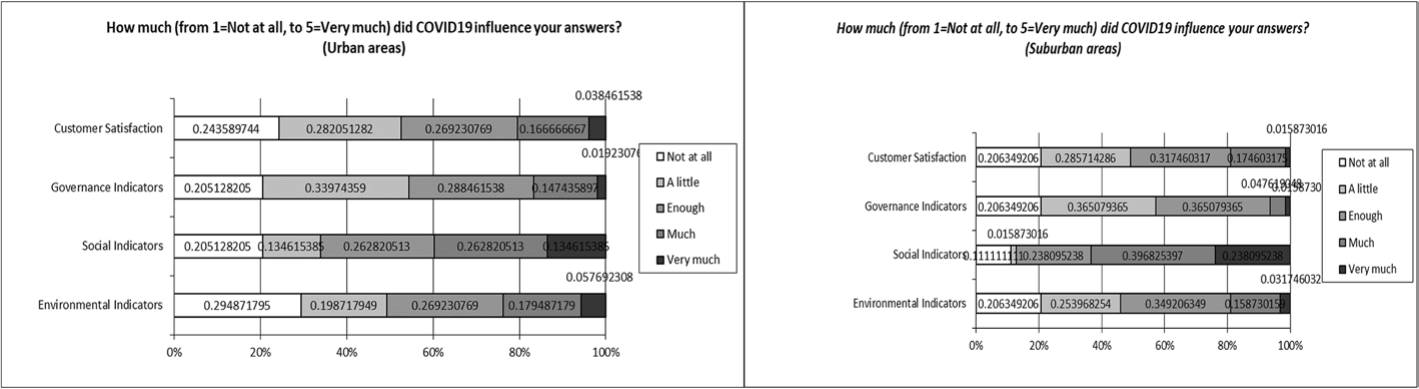
Graphical representation of Impact of COVID-19 on the impact of ESG indicators

Regarding the second research question, Table 7 and Fig. 7 below presents the main survey descriptive results (mean, standard deviation, and graphical representation), which show that respondents’ perceptions of the impact of ESG indicators on their decision to purchase products/services from a company operating in Greece, as well as policies implementing these indicators, appear to be largely unaffected by COVID-19. However, it is worth noting that the greatest impact of COVID-19 is shown in the “social indicators,” a result that is in line with the research of Mitra and Anas [53]. This result may be due to the increase in social awareness of citizens, as the main negative effects of the pandemic occurred in areas of social well-being.

**Table 7:** Impact of COVID-19 on the impact of ESG indicators

*How much (from 1* = *not at all, to 5* = *very much) did COVID19 influence your answers?*	Sample	1 = not at all	2 = a little	3 = enough	4 = much	5 = very much	Average	Std. Dev
Environmental indicators	Urban	46	31	42	28	9	2,51	1,25
	Suburban	26	32	44	20	4	2,56	1,08
Social indicators	Urban	32	21	41	41	21	2,99	1,33
	Suburban	14	2	30	50	30	3,63	1,19
Governance indicators	Urban	32	53	45	23	3	2,44	1,04
	Suburban	26	46	46	6	2	2,30	0,91
Customer satisfaction	Urban	38	44	42	26	6	2,47	1,14
	Suburban	26	36	40	22	2	2,51	1,06

In order to study the third research question and to determine whether the variables depend on the demographic characteristics of the sample, the following statistical analysis was carried out:

- Mann–Whitney *U* test for gender and employment status differences (employed: entrepreneur/freelance, private employee, public employee; non-employed: student/student, unemployed, homemaker, retired)
- Kruskal–Wallis test, for age, monthly income, and educational level differences (as these are classifiable variables)

For conducting the tests, we consider that the null hypothesis Η_0_ assumes independence and the alternative of Η_1_ assumes their dependence. The hypotheses are tested at the 5% level of significance.

As can be seen in Table 8 below, with respect to gender (male–female):

**Table 8:** Correlation test for gender (Mann–Whitney *U* test)

		Urban areas (inside Attica region)			Suburban areas (outside Attica region)		
	Gender	Average	Mann–Whitney *U* test	*p*-value	Average	Mann–Whitney *U* test	*p*-value
Environmental indicators	Men	3.67	2,966,000	0.807	4.23	1,378,000	0.005
	Women	3.76			3.79		
Social indicators	Men	3.75	2,359,500	0.017	4.03	1,844,000	0.621
	Women	4.10			3.93		
Governance indicators	Men	3.39	2,609,000	0.129	3.06	1,942,000	0.992
	Women	3.64			3.08		
Customer satisfaction	Men	3.66	2,470,500	0.043	3.83	1,792,000	0.445
	Women	3.99			3.71		

For the urban sample, it can be seen that:

- The Η_0_ hypothesis is accepted for the variables “environmental indicators” and “corporate governance indicators” as *p*-value (sig.) > 0.05. That is, the above mentioned variables are not dependent on the gender of the sample.
- Hypothesis Η_1_ is accepted for the variables “social indicators” and “customer satisfaction,” as *p*-value < 0.05; i.e., the aforementioned variables are dependent on the gender of the sample, with females, scoring higher on the individual factors surrounding these variables.

For the suburban sample, it follows that:

- Hypothesis Η_0_ is accepted for the variables “social indicators,” “corporate governance indicators,” and “customer satisfaction” as *p*-value > 0.05. That is, the aforementioned variables are not dependent on the gender of the sample.
- Hypothesis Η_1_ is accepted for the variable “environmental indicators” as *p*-value < 0.05; i.e., this variable depends on the gender of the sample, with males scoring higher on the individual factors surrounding this variable.

As can be seen in Table 9 below, in terms of employment status (employed—non-employed), where employed: entrepreneur/freelance, private employee, public employee and non-employed: student, unemployed, homemaker, pensioner:

**Table 9:** Correlation test for employment status (Mann–Whitney *U* test)

		Urban areas			Suburban areas		
	Employment status	Average	Mann–Whitney *U* test	*p*-value	Average	Mann–Whitney *U* test	*p*-value
Environmental indicators	Employed	3.81	2,626,000	0.175	4.00	1,792,000	0.685
	Non employed	3.60			4.11		
Social indicators	Employed	4.06	2,233,500	0.006	3.99	1,848,000	0.904
	Non employed	3.73			3.99		
Governance indicators	Employed	3.68	2,216,000	0.005	3.12	1,750,000	0.537
	Not employed	3.29			2.98		
Customer satisfaction	Employed	3.97	2,136,000	0.002	3.84	1,778,000	0.544
	Non employed	3.62			3.69		

a) for the urban sample, it appears that:

- Hypothesis Η_0_ is accepted for the variable “environmental indicators” as *p*-value > 0.05. This means that the above variable does not depend on the employment status of the sample.
- Hypothesis Η_1_ is accepted for the variables “social indicators,” “corporate governance indicators,” and “customer satisfaction,” as *p*-value < 0.05 (dependence of these variables on employment status). More specifically, it is observed that the survey participants who are employees score higher on the individual factors surrounding the aforementioned variables, compared to non-employees.

b) for the suburban sample, it can be seen that:

- Hypothesis Η_0_ is accepted for all variables of the research model as *p*-value > 0.05. That is, the above variables do not depend on the employment status of the sample.

As can be seen in Table 10 below, with regard to age:

**Table 10:** Correlation test by age (Kruskal–Wallis *X*^2^ test)

		Urban areas			Suburban areas		
	Age	Average	Kruskal–Wallis *X*^2^ test	*p*-value	Average	Kruskal–Wallis *X*^2^ test	*p*-value
Environmental indicators	18–25	3.73	4421	0.219	4.37	18,571	< 0.001
	26–45	3.75			4.24		
	46–66	3.83			3.69		
	66 +	3.31			3.84		
Social indicators	18–25	3.73	6797	0.079	4.10	14,926	0.002
	26–45	4.05			4.20		
	46–66	4.00			3.69		
	66 +	3.59			3.99		
Governance indicators	18–25	3.58	2104	0.551	3.13	10,881	0.012
	26–45	3.56			3.28		
	46–66	3.47			2.87		
	66 +	3.29			2.60		
Customer satisfaction	18–25	3.77	3870	0.276	3.83	10,363	0.016
	26–45	3.96			3.94		
	46–66	3.75			3.68		
	66 +	3.61			3.30		

a) for the urban sample, it can be seen that:

- Hypothesis Η_0_ is accepted for all variables in the research model as *p*-value > 0.05. That is, the above variables do not depend on the age of the urban sample.

b) for the suburban sample, it follows that:

- Hypothesis Η_1_ is accepted for all variables in the research model, as *p*-value < 0.05 (i.e. dependence of all variables on the age of the sample). It is observed that the younger respondents of the suburban sample scored higher on the factors surrounding the variables of the research model (or vice versa it is observed that as the age of the respondents increases, the mean value of the variables of the research model decreases).

By the inspection of Table 11, regarding income, it can be seen that the Η_0_ hypothesis is accepted for all variables in the research model as *p*-value > 0.05. That is, the above variables do not depend on the monthly income of both samples.

**Table 11:** Correlation test based on income (Kruskal–Wallis *X*^2^ test)

		Urban areas			Suburban areas		
	Income	Average	Kruskal–Wallis *X*^2^ test	*p*-value	Average	Kruskal–Wallis *X*^2^ test	*p*-value
Environmental indicators	up to 1000 €	3.61	5344	0.069	3.99	0.375	0.909
	1000–1800 €	3.71			4.08		
	1800 € and over	4.22			3.97		
Social indicators	up to 1000 €	3.81	2539	0.281	3.97	0.350	0.840
	1000–1800 €	4.06			4.04		
	1800 € and over	4.05			3.91		
Governance indicators	up to 1000 €	3.41	4304	0.116	2.99	3.808	0.149
	1000–1800 €	3.58			3.15		
	1800 € and over	3.82			3.02		
Customer satisfaction	up to 1000 €	3.77	3854	0.146	3.82	1.674	0.433
	1000–1800 €	4.00			3.83		
	1800 € and over	3.62			3.90		

As can be seen in Table 12 below, with regard to education level, it can be seen that hypothesis Η_1_ is accepted for all variables in the research model, as *p*-value < 0.05 (i.e., dependence of all variables on the education level of both samples). It is observed that as the educational level of the two samples increases, the mean value of the variables under study also increases.

**Table 12:** Correlation test based on education (Kruskal–Wallis *X*^2^ test)

		Urban areas			Suburban areas		
	Education	Average	Kruskal–Wallis *X*^2^ test	*p*-value	Average	Kruskal–Wallis *X*^2^ test	*p*-value
Environmental indicators	Secondary	3.20	17,983	< 0.001	3.42	36,776	< 0.001
	Tertiary	3.92			4.23		
	Postgraduate/doctoral	3.90			4.44		
Social indicators	Secondary	3.54	13,784	0.001	3.40	33,516	< 0.001
	Tertiary	4.06			4.24		
	Postgraduate/doctoral	4.08			4.24		
Governance indicators	Secondary	3.16	12,080	0.002	2.36	50,675	< 0.001
	Tertiary	3.61			3.28		
	Postgraduate/doctoral	3.70			3.53		
Customer satisfaction	Secondary	3.58	7,814	0.020	3.34	24,823	< 0.001
	Tertiary	3.91			3.91		
	Postgraduate/doctoral	3.92			4.07		

A correlation analysis was also performed between the “environmental indicators,” “social indicators,” “corporate governance indicators,” and “customer satisfaction,” as shown in Table 13, yielding the following results for the urban sample:

- Strong positive correlation between “customer satisfaction” and “corporate governance indicators,” at a statistical significance level of 0.01 (Spearman’s rho = 0.696; *p*-value < 0.001). That is, as the levels of “corporate governance indicators” increase, the levels of customer satisfaction will increase.
- Strong positive correlation between “customer satisfaction” and “social indicators,” at a statistical significance level of 0.01 (Spearman’s rho = 0.720; *p*-value < 0.001). That is, as the levels of “social indicators” increase, so will the levels of customer satisfaction.
- Strong positive correlation between “customer satisfaction” and “environmental indicators,” at a statistical significance level of 0.01 (Spearman’s rho = 0.597; *p*-value < 0.001). That is, as the levels of “environmental indicators” increase, so will the levels of customer satisfaction.
- Strong positive correlation between “corporate governance indicators” and “social indicators,” at a statistical significance level of 0.01 (Spearman’s rho = 0.723; *p*-value < 0.001). That is, as the levels of “social indicators” increase, the levels of “corporate governance indicators” will also increase.
- Strong positive correlation between “corporate governance indicators” and “environmental indicators,” at a statistical significance level of 0.01 (Spearman’s rho = 0.775; *p*-value < 0.001). That is, as the levels of the “environmental indicators” increase, so will the levels of the “corporate governance indicators.”
- Strong positive correlation between “social indicators” and “environmental indicators,” at a statistical significance level of 0.01 (Spearman’s rho = 0.663; *p*-value < 0.001). That is, as the levels of the “environmental indicators” increase, so will the levels of the “social indicators.”

**Table 13:** Correlation analysis results (Spearman’s rho)

		Urban areas				Suburban areas			
	Spearman’s rho	Env	Soc	Gov	Cust	Env	Soc	Gov	Cust
Environmental indicators	Correlation coefficient	1				1			
	*p*-value (2-tailed)								
	*N*	156				126			
Social indicators	Correlation coefficient	0.633^*^	1			0.665^*^	1		
	*p*-value (2-tailed)	< 0.001				< 0.001			
	*N*	156	156			126	126		
Governance indicators	Correlation coefficient	0.775^*^	0.723^*^	1		0.448^*^	0.533^*^	1	
	*p*-value (2-tailed)	< 0.001	< 0.001			< 0.001	< 0.001		
	*N*	156	156	156		126	126	126	
Customer satisfaction	Correlation coefficient	0.597^*^	0.720^*^	0.696^*^	1	0.533^*^	0.486^*^	0.406^*^	1
	*p*-value (2-tailed)	< 0.001	< 0.001	< 0.001		< 0.001	< 0.001	< 0.001	
	*N*	156	156	156	156	126	126	126	126

*Correlation is significant at the 0.01 level (2-tailed)

For the suburban sample, it follows:

- Positive correlation between “customer satisfaction” and “corporate governance indicators,” at a statistical significance level of 0.01 (Spearman’s rho = 0.406; *p*-value < 0.001). That is, as the levels of “corporate governance indicators” increase, the levels of customer satisfaction will also increase.
- Positive correlation between “customer satisfaction” and “social indicators,” at a statistical significance level of 0.01 (Spearman’s rho = 0.486; *p*-value < 0.001). That is, as the levels of “social indicators” increase, the levels of customer satisfaction will also increase.
- Positive correlation between “customer satisfaction” and “environmental indicators”, at a statistical significance level of 0.01 (Spearman’s rho = 0.533; *p*-value < 0.001). That is, as the levels of “environmental indicators” increase, so will the levels of customer satisfaction.
- Positive correlation between “corporate governance indicators” and “social indicators,” at a statistical significance level of 0.01 (Spearman’s rho = 0.533; *p*-value < 0.001). That is, as the levels of “social indicators” increase, the levels of “corporate governance indicators” will also increase.
- Positive correlation between “corporate governance indicators” and “environmental indicators,” at a statistical significance level of 0.01 (Spearman’s rho = 0.448; *p*-value < 0.001). That is, as the levels of the “environmental indicators” increase, so will the levels of the “corporate governance indicators.”
- Strong positive correlation between “social indicators” and “environmental indicators,” at a statistical significance level of 0.01 (Spearman’s rho = 0.665; *p*-value < 0.001). That is, as the levels of the “environmental indicators” increase, so will the levels of the “social indicators.”

Finally, in order to investigate the fourth research question, “customer satisfaction” was estimated using the multiple linear regression method, with the “environmental indicators,” “social indicators,” and “corporate governance indicators” serving as independent variables (predictors). First, a reliability analysis was performed using Cronbach’s alpha and the internal consistency method. The Cronbach’s alpha index is greater than the allowable limits set for all variables, as shown in Table 14. As a result, the individual variables that comprise the variables measure the same research item, and the scales used to measure the variables are reliable.

**Table 14:** Internal consistency control results (Cronbach’s alpha)

Cronbach’s alpha	Urban areas	Suburban areas	Items
Environmental indicators	0.974	0.939	5
Social indicators	0.944	0.932	9
Governance indicators	0.946	0.902	4
Customer satisfaction	0.922	0.897	4

Tables 15 and 16 show the estimation results for the urban area and suburban area samples, revealing that variations in the dependent variable are explained by variations in the independent variables by 59.7% and 41.4%, respectively. Specifically, Table 15 shows the goodness-of-fit results for the two regression models, whereas Table 16 presents the parameter estimate results. The independent variables that satisfy the statistical significance condition of 5% *p*-value and can predict the dependent variable are as follows:

**Table 15:** Regression modeling goodness-of-fit

Model	*R*	*R*^*2*^	Adjusted *R*^*2*^	Std. error of the estimate
Urban areas	0.773	**0.597**	0.589	0.526
Suburban Areas	0.643	**0.414**	0.399	0.534

**Table 16:** Regression modeling Summary (parameter estimates along with statistical significance)

	Urban area (inside Attica region)				Suburban area (outside Attica region)			
	Coefficients		*t*	*p*-value	Coefficients		*t*	*p*-value
	*B*	Std. error			*B*	Std. error		
Constant	0.951	0.197	4.823	< 0.001	1.245	0.279	4.459	< 0.001
Environmental indicators	0.008	0.070	0.117	0.907	0.258	0.086	3.016	0.003
Social indicators	0.455	0.079	5.788	< 0.001	0.280	0.101	2.771	0.006
Governance indicators	0.301	0.092	3.269	0.001	0.122	0.071	1.720	0.088

*Dependent variable: customer satisfaction; predictors: (constant), governance indicators, environmental indicators, social indicators*

For the sample residing within the urban area:

- “Social indicators” (*p*-value < 0.001), which change in the same direction as the dependent variable (Beta =  + 0.482 > 0)
- “Corporate governance indicators” (*p*-value = 0.001), which vary in the same direction as the dependent variable (Beta =  + 0.328 > 0)

For the sample residing in the suburban area:

- “Environmental indicators” (*p*-value = 0.003), which change in the same direction as the dependent variable (Beta =  + 0.305 > 0)
- “Social indicators” (*p*-value = 0.006), which vary in the same direction as the dependent variable (Beta =  + 0.296 > 0)

## Discussion and Conclusions

As pointed in ATHEX (2019) [7], effective management of ESG issues can bring significant benefits to companies, the main ones being:

- *Improved access to capital.* Investment decisions, particularly those made by institutional investors, necessitate the incorporation of ESG data. As a result, a company’s ability to attract investors is enhanced on the one hand by transparency about its performance, and on the other by how ESG issues are managed in the creation of long-term value. Companies that effectively disclose non-financial information and demonstrate good performance on ESG issues, according to Cheng, Ioannou, and Serafeim [43], have a greater ability to access capital at lower costs.
- *Compliance with regulatory changes.* In terms of sustainable development, an increasing number of governments are adopting the 2014/95/EU Directive on non-financial information disclosure by companies. As a result, disclosure of non-financial corporate information is a legal requirement. According to Grewal, Riedl, and Serafeim [44], companies that establish ESG information disclosure procedures will be able to respond and comply more effectively to external regulatory, legislative, and legal changes, ensuring their licensed operation.
- *Enhancing corporate performance.* Research links higher corporate performance (stock return, profitability, business results) with good performance on key ESG indicators that contribute to long-term value generation [45].
- *Enhancing corporate reputation and stakeholder engagement.* Disclosing non-financial information and improving a company’s ESG performance demonstrates its commitment to transparency best practices, alignment with sustainable development goals, and long-term value creation [46]. The aforementioned corporate attitude, which improves corporate reputation, is communicated to stakeholders, providing increasingly meaningful opportunities to engage with them [46].

This study found that ESG indicators, with a focus on environmental and social indicators, have a moderate to high influence on consumers’ decisions to choose products/services based on the policies implemented regarding these indicators, regardless of their region of residence (urban and suburban area). The pandemic does not appear to have changed their minds significantly, though it should be noted that it had a greater impact on social indicators, which is consistent with Mitra and Anas’ findings [38]. This result may be due to the increase in social awareness of citizens as the main negative effects of the pandemic occurred in areas of social well-being. Also, many companies during the COVID 19 period recognized necessity of disclosing information and indicators on additional to already existing social issues [47].

Social indicators seem to have a greater influence on the choices of the urban sample, while for the suburban sample, the environmental indicators play a more important role. Governance indicators for both samples had no significant effect on their choices, with the urban sample rating them higher than the sample residing outside. The high scores given to the environmental indicators by the suburban sample may be due to the fact that the economic development of these local communities is directly linked to the environment (agriculture, tourism related to the environment) and therefore the policies for its protection, implemented by the companies, are an important criterion for their choice of purchase for this sample. These results may provide indications and suggestions to both urban and suburban companies in order to improve their resilience by taking into consideration these findings relating to the customer choices and preferences as these are found to be affected by the three ESG indicators.

The following results were obtained from the test of dependence of the sample’s demographic data on the variables of the research model:

- Women in the sample who live in cities performed better on the “social indicators.” This finding could be explained by the fact that women face more injustice/discrimination in terms of professional rehabilitation as well as prejudices about women’s roles in society.
- Employees in the sample who lived in urban areas performed better on “social indicators” and “corporate governance indicators.”
- This result may be based on the fact that citizen employees perceive to a greater extent (insider experience) that the financial performance of a company depends to a significant extent on activities related to society, human rights, labor relations, and business ethics.
- The younger respondents of the suburban sample rated higher the factors framing all variables of the research model. Younger age respondents who do not reside in a large urban center may be more concerned to a higher degree about the social, environmental, and ethical problems of business activities, the non-solution of which is the source of the need for these individuals to move to large urban centers.
- Higher educated individuals in both samples scored higher on all ESG indicators. It is possible that the education and information style (as it fits the higher educated profile) of these individuals may reinforce perceptions linking a company’s financial performance to ESG indicators.

Furthermore, no significant COVID-19 effect was evidenced on the findings, although the emphasis on “social indicators” was further reinforced during the pandemic, probably due to the increase in social awareness of citizens.

Finally, a positive correlation was found between the dependent variable “customer satisfaction” (customer retention and spreading positive word of mouth feedback [13]) and the independent variables “environmental indicators,” “social indicators,” and “governance indicators” from the correlation analysis and subsample estimation for both samples. Those are consistent with the findings of the research of Saleh, Ebeid, and Abdelhameed [13], Cek and Eyupoglu [24], Lubowiecki-Vikuk et al. [20], and Mitra and Anas [38]. For the sample residing within urban regions, the independent variables that can predict the dependent variable are “social indicators” and “governance indicators.” The findings of Cek and Eyupoglu [24] showed that performance on ESG indicators significantly affect the financial performance of the companies under consideration (financial performance is directly dependent on customer satisfaction) with social and corporate governance indicators being the most critical, compared to environmental indicators. For the suburban sample, the independent variables that can predict the dependent variable are “social indicators” and “environmental indicators.” It becomes clear that the social factor is present in both samples, with the urban sample region placing more emphasis on corporate governance issues, while the suburban sample places more emphasis on environmental issues.

To make the findings more widely applicable, the current work could be expanded by the following:

- Examining an even larger sample from all regions of the country in order to assess any regional differences between them
- Studying the existence of any differences in the importance of ESG for the choice of consumption/buying products/services of different sectors of economic activity
- To be repeated after a few years in order to identify and comment on changes linked to c (e.g., similar to the COVID pandemic)

## Data Availability

Collected data are available upon request by the corresponding author.

## Abbreviations

ESG: environmental-social-governmental
